# Neuroprotective effects of bajijiasu against cognitive impairment induced by amyloid-β in APP/PS1 mice

**DOI:** 10.18632/oncotarget.21515

**Published:** 2017-10-04

**Authors:** Haobin Cai, Yijie Wang, Jiayang He, Tiantian Cai, Jun Wu, Jiansong Fang, Rong Zhang, Zhouke Guo, Li Guan, Qinkai Zhan, Li Lin, Yao Xiao, Huafeng Pan, Qi Wang

**Affiliations:** ^1^ Institute of Clinical Pharmacology, Guangzhou University of Chinese Medicine, Guangzhou 510405, China; ^2^ Department of Neurology & Psychology, Shenzhen Hospital Affiliated to Guangzhou University of Chinese Medicine, Shenzhen 518033, China; ^3^ Guangzhou University of Chinese Medicine, Guangzhou 510405, China; ^4^ Guangzhou Medical University, Guangzhou 510182, China

**Keywords:** dementia, bajijiasu, amyloid-β, cognitive impairment, APP/PS1 mice

## Abstract

Alzheimer's disease (AD) is a progressive neurological degenerative disease. The main clinical manifestations of AD include progressive cognitive impairment and alteration of personality. Senile plaques, neuroinflammation, and destruction of synapse structure stability are the main pathological features of AD. Bajijiasu(BJJS) is extracted from Morinda Officinalis, a Chinese herb. In this study, we explored the effect of BJJS on AD from many aspects in APPswe/PSEN1ΔE9 (APP/PS1) double transgenic mice. The Morris water maze and novel object recognition tests results showed that BJJS could significantly improve the learning and memory abilities in APP/PS1 mice. BJJS treatment increased the level of insulin degradation enzyme (IDE) and neprilysin (NEP) and decreased the level of β-site app cleaving enzyme 1(BACE1) in the brain of APP/PS1 mice. BJJS-treated APP/PS1 mice appeared to have reductions of Aβ deposition and senile plaques, and showed higher levels of neurotrophic factors in the brain. We also found that BJJS had an inhibitory function on neuroinflammation in APP/PS1 mice. In addition, the synapse structure relevant proteins were elevated in the brain of BJJS-treated APP/PS1 mice. The present results indicated that BJJS could attenuate cognitive impairment via ameliorating the AD-related pathological alterations in APP/PS1 mice. These findings suggest that BJJS may be a potential therapeutic strategy in Alzheimer's disease.

## INTRODUCTION

As we know, Alzheimer's disease (AD) is the most common dementia among the aged population. Memory impairment and progressive cognitive dysfunction are the most representative symptoms of AD [1]. The pathological hallmarks of AD by the presence of extracellular senile plaques composed of accumulation and aggregation of amyloid-β (Aβ) peptide, intraneuronal neurofibrillary tangles (NFTs), and chronic neuroinflammation [2]. Numerous studies have focused on the neurotoxicity of Aβ since they were identified in senile plaques from AD patients decades ago [3, 4]. Aβ peptides are derived from the sequential proteolysis of the amyloid precursor protein (APP), a single-pass transmembrane glycoprotein with a large extracellular domain. Firstly, the amyloidogenic processing of APP is mainly catalyzed by β-site app cleaving enzyme 1 (BACE1), the canonical β-secretase. This processing leads to the shedding of the ectodomain (soluble APP-β fragment or sAPP-β) and the production of a small membrane-spanning C-terminal fragment β (CTF-β). Then CTF-β is further cleaved by γ-secretase in the transmembrane domain, and finally generating Aβ. The C-terminal fragment γ is released into the cytoplasm [5]. APP and presenilin 1 (PS1) genes are respectively associated with increased amyloidogenic processing of APP and preferential production of longer Aβ species with higher amyloidogenic propensity, such as Aβ1-42, the most neurotoxic amyloid fragment [6]. A plethora of mouse models of Aβ pathology have been established through several of these mutations [7]. Nevertheless, none of them could fully simulate the cognitive impairment and pathological process described in AD patients [8]. As in the animal model used in this study, overexpression of APP or PS1 poses several drawbacks, such as overproduction of APP and its cleavage fragments besides Aβ. This also has an impact on AD-associated phenotypes [9, 10]. However, APP/PS1 double transgenic mice are among the most successful models, promptly developing memory and cognitive impairments and other relevant pathological process, such as Aβ deposition and a robust neuroinflammatory response toward senile plaques, along with synaptic integrity loss [11]. The precise molecular mechanism by which Aβ exerts its toxicity is still unclear; however, Aβ is thought to be one of the key contributors to the chronic inflammatory response in the AD brain. Aβ accumulation and aggregation can elicit the development of inflammatory processes and activate microglia and reactive astrocytes in the vicinity of senile plaques [12]. During the progression of AD, sustained glial activation increases the levels of secreted proinflammatory molecules. In addition, these inflammatory processes can induce their own expression in a feedback loop. These interactions further exacerbate pathological process of AD, finally contributing to neurodegeneration and dysregulation of signaling pathways that favor the amyloidogenic process of APP [13].

Neurotrophic factors play crucial roles in protecting the peripheral and central nervous systems. Neurotrophic factors promote the growth, development and survival of neurons, prevent neurons from damage, fight against the neurotoxicity and neuroinflammation caused by amyloid-β deposition, induce and maintain the presynaptic and postsynaptic long-term potentiation (LTP), and also participate in the process of hippocampus-dependent learning and memory [14, 15].

BJJS, which is isolated from Morinda Officinalis, is able to reinforce population spikes (PSs) and long-term potentiation (LTP) [16], attenuate cognitive impairments caused by D-galactose in mice, and protect against ischemia-induced neuronal damage or death in previous studies [17, 18]. It has been revealed that BJJS protected PC12 cells from Aβ25-35 induced neurotoxicity in cell culture experiments [19]. What‘s more, a recent article discussed the neuroprotective effects of BJJS, which were shown in the rat model of Aβ25-35 induced neurotoxicity, and analyzed the chemical structure of BJJS [20].

The objective of this study was to investigate the neuroprotective effects of BJJS on APP/PS1 double transgenic mice and explore the underlying mechanisms of BJJS *in vivo*. We propose a research hypothesis that BJJS may improve the learning and memory abilities of APP/PS1 mice via regulating the metabolism of amyloid-β and affecting many other factors associated with AD in the brain. Our study was to confirm that BJJS may serve as a promising therapeutic drug for Alzheimer's disease.

## RESULTS

### BJJS improved cognitive performance in APP/PS1 mice

In the present study, Morris water maze and novel object recognition tests were used to evaluate the effects of BJJS on learning and memory abilities in APP/PS1 mice.

**Figure 1:**
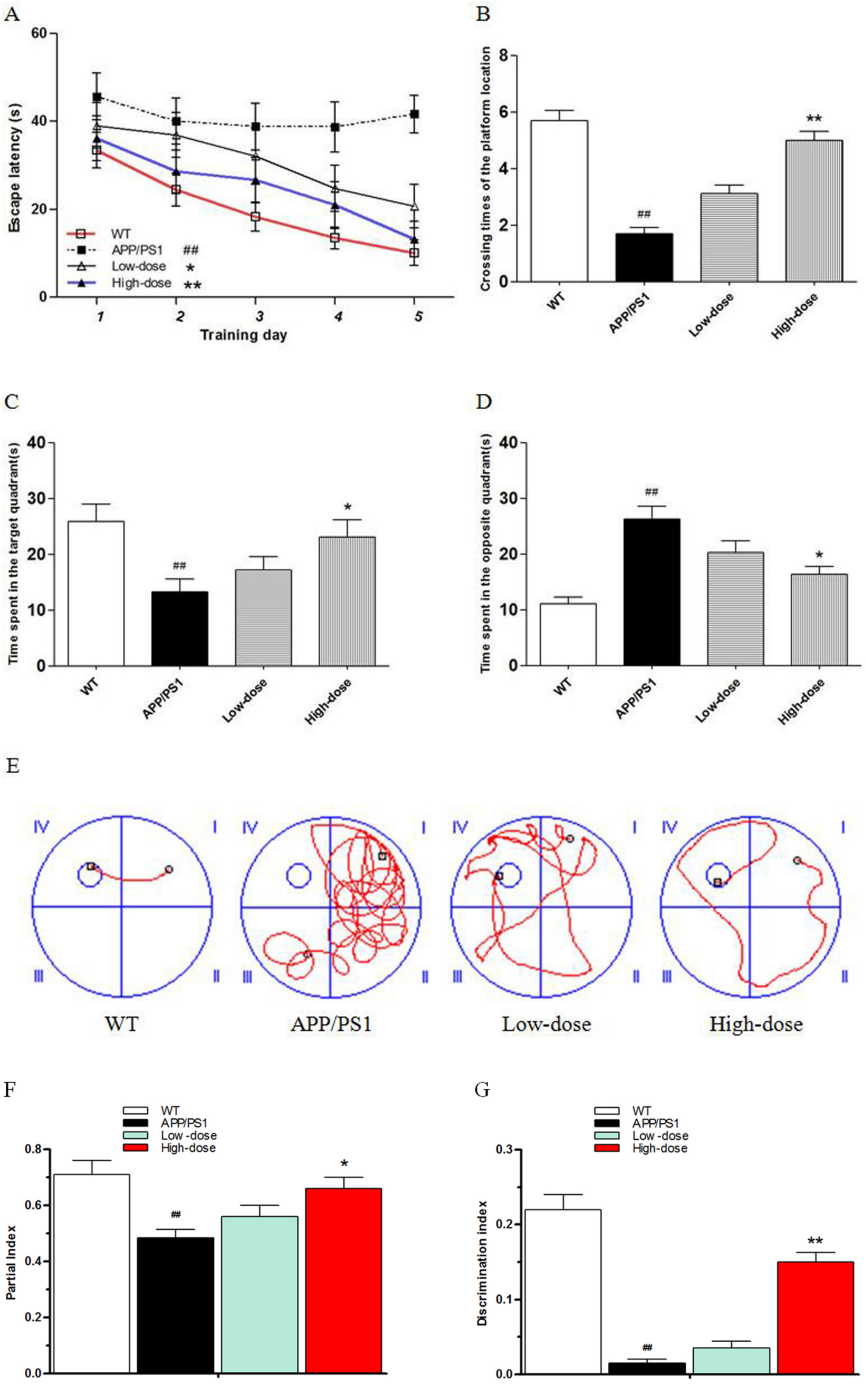
BJJS improved the learning and memory abilities in APP/PS1 mice **(A)** Escape latencies in water maze during the positioning navigation test. **(B)** Crossing times of the platform location during the probe trial. **(C, D)** Time spent in the target quadrant and in the opposite quadrant during the probe trial. **(E)** Representative tracings of the animal's path during the positioning navigation test. **(F)** The partial index on the third day of the novel object recognition test. **(G)** The discrimination index on the third day of the novel object recognition test. Data are shown as the mean ± SEM (n=10 mice per group). ^#^ *P*<0.05 and ^##^ *P*<0.01 versus the wild-type group. ^*^ *P*<0.05 and ^**^ *P*<0.01 versus the APP/PS1 group.

In the Morris water maze test, the time of the mice to reach the hidden platform among the 4 experimental groups were compared (Figure 1A). In the 5-days positioning navigation test, the escape latency of the APP/PS1 group significantly prolonged compared with the wild-type group (*P*<0.01 vs. wild-type) (Figure 1A). The escape latency of BJJS low-dose and high-dose groups performed shorter compared with the APP/PS1 group during the 5-days positioning navigation test (*P*<0.05, *P*<0.01, respectively vs. APP/PS1). Figure 1E was the representative tracings figures of the animal's path on the 5^th^ day of the positioning navigation test. On the 7^th^ day of MWM test, the platform was removed to do the probe test. The result revealed that the crossing times of the platform location and the time spent in the target quadrant or in the opposite quadrant significantly differed among the 4 experimental groups (Figure 1B, 1C, 1D). The APP/PS1 group significantly crossed less times compared with the wild-type group (*P*<0.01 vs. wild-type) (Figure 1B). The high-dose group significantly crossed more times compared with the APP/PS1 group (*P*<0.01 vs. APP/PS1) (Figure 1B). More importantly, the APP/PS1 group significantly spent less time in the target quadrant but more time in the opposite quadrant compared with the wild-type group (*P*<0.01 vs. wild-type) (Figure 1C, 1D). The high-dose group also significantly spent more time in the target quadrant but less time in the opposite quadrant compared with the APP/PS1 group (*P*<0.05 vs. APP/PS1) (Figure 1C, 1D).

In the novel object recognition test, the mice were allowed to have a freedom activity in the test box for 5 min on the first day for acclimatization. Then, the mice were exposed to the box with 2 same objects on the second day, and there was no significant difference in partial index between any two experimental groups (*P*>0.05). However, when mice were exposed to the box with one of the objects replaced with a new one on the third day, there was no significant difference in the exploration of APP/PS1 and low-dose mice to two different objects. The partial index in the APP/PS1 group was significantly lower compared with the wild-type group (*P*<0.01 vs. wild-type) (Figure 1F). The partial index in the high-dose group was significantly higher compared with the APP/PS1 group (*P*<0.05 vs. APP/PS1) (Figure 1F). Then, we analyzed the discrimination index (novel object exploration minus familiar object exploration / total exploration time) of the mice. As expected, the APP/PS1 group perform significantly lower discrimination index compared with the wild-type group (*P*<0.01 vs. wild-type) (Figure 1G). The discrimination index of the high-dose group was significantly higher compared with the APP/PS1 group (*P*<0.01 vs. APP/PS1) (Figure 1G).

The MWM and NORT tests results indicated that BJJS could enhance the learning and memory abilities in APP/PS1 mice.

### BJJS regulated the metabolism of amyloid-β in APP/PS1 mice

At 6 weeks of age, APP/PS1 mice beginning to show increased Aβ generation and Aβ deposition in the cortex, and in the hippocampus at 2∼3 months of age [21]. In the present study, we evaluated the effect of BJJS on the metabolism of amyloid-β in APP/PS1 mice after the behavior tests.

**Figure 2:**
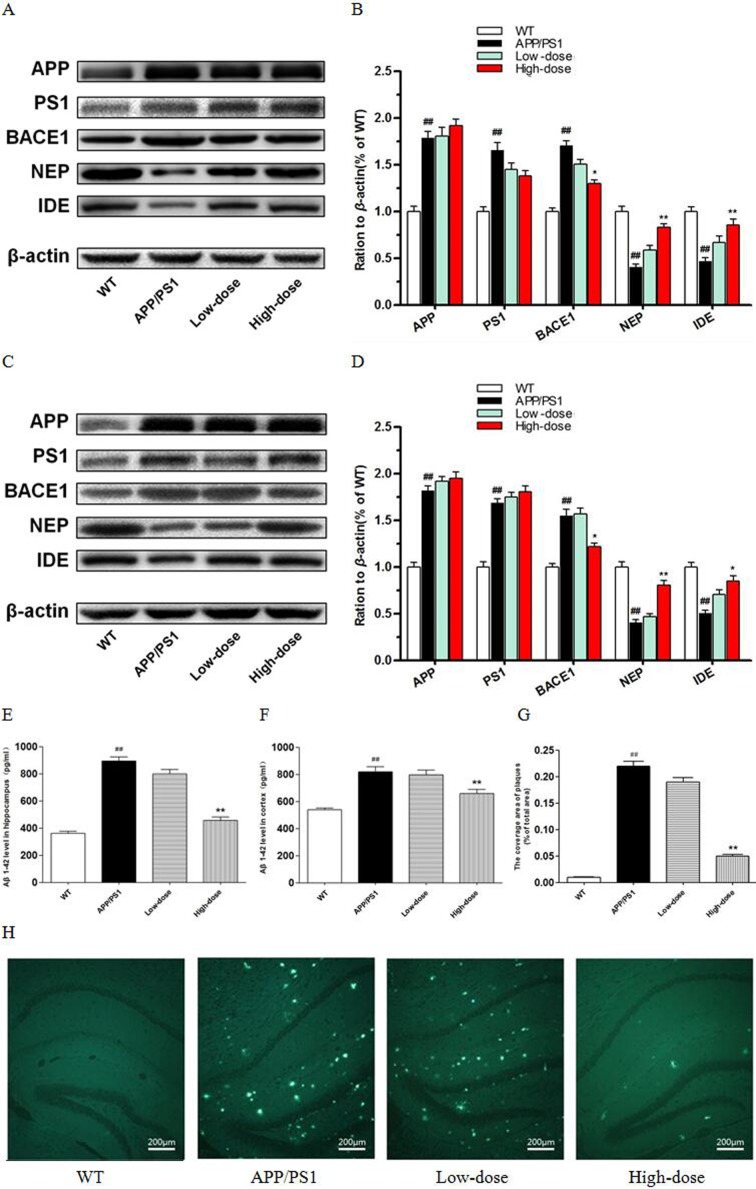
BJJS attenuated amyloid-β generation and deposition in APP/PS1 mice **(A, B)** The levels of proteins associated with amyloid-β metabolism in the hippocampus. **(C, D)** The levels of proteins associated with amyloid-β metabolism in the cortex. **(E)** The level of Aβ1-42 in the hippocampus. **(F)** The level of Aβ1-42 in the cortex. **(G, H)** Representative images and quantitative graph for Thioflavin-S staining in the brain of APP/PS1 mice. Data are shown as the mean ± SEM (n=10 mice per group). ^#^ *P*<0.05 and ^##^ *P*<0.01 versus the wild-type group. ^*^ *P*<0.05 and ^**^ *P*<0.01 versus the APP/PS1 group.

To evaluate the effects of treatment with BJJS on the metabolism of amyloid-β, the protein levels of amyloid precursor protein (APP), presenilin 1(PS1), β-site app cleaving enzyme 1(BACE1), insulin degradation enzyme (IDE) and neprilysin (NEP) were analyzed by western blot (Figure 2A, 2B, 2C, 2D). The wild-type mice showed a higher level of APP compared with the APP/PS1 group (*P*<0.01 vs. wild-type) (Figure 2A, 2B, 2C, 2D). The level of APP in BJJS groups changed insignificantly compared with the APP/PS1 group (*P*>0.05 vs. APP/PS1) (Figure 2A, 2B, 2C, 2D), and the detected result of PS1 was similar as APP (Figure 2A, 2B, 2C, 2D). The noteworthy was that the high-dose BJJS significantly reduced the level of BACE1 both in the hippocampus and cortex compared with the APP/PS1group (*P*<0.05 vs. APP/PS1) (Figure 2A, 2B, 2C, 2D). What's more, the high-dose BJJS remarkably enhanced the expression of NEP (*P*<0.01 vs. APP/PS1) (Figure 2A, 2B, 2C, 2D) and IDE (*P*<0.05 vs. APP/PS1) (Figure 2A, 2B, 2C, 2D) both in the hippocampus and cortex compared with the APP/PS1 group.

In addition, we also used Enzyme-linked immune sorbent assay to evaluate the level of Aβ1-42 both in hippocampus and cortex of the 4 experimental groups (Figure 2E, 2F). As expected, the APP/PS1 group presented a significantly higher level of Aβ1-42 compared with the wild-type group (*P*<0.01 vs. wild-type) (Figure 2E, 2F). Interestingly, we found that the high-dose BJJS significantly decreased the level of Aβ1-42 both in the hippocampus and cortex compared with the APP/PS1 group (*P*<0.01 vs. APP/PS1) (Figure 2E, 2F).

Then, we used Thioflavine-S (Th-S) staining to further confirm the effect of BJJS on the deposition and accumulation of amyloid-β. Thioflavine-S (Th-S) staining (Figure 2G, 2H) revealed the presence of extensive senile plaques in the brain of APP/PS1 mice (*P*<0.01 vs. wild-type) (Figure 2G, 2H). What's more, the number of senile plaques was significantly decreased in the brain of high-dose BJJS group compared with the APP/PS1 group (*P*<0.01 vs. APP/PS1) (Figure 2G, 2H).

All these results indicated that BJJS may mitigate the amyloid-β generation and deposition by mediating the metabolism of amyloid-β.

### BJJS enhanced the expression of neurotrophic factors in APP/PS1 mice

**Figure 3:**
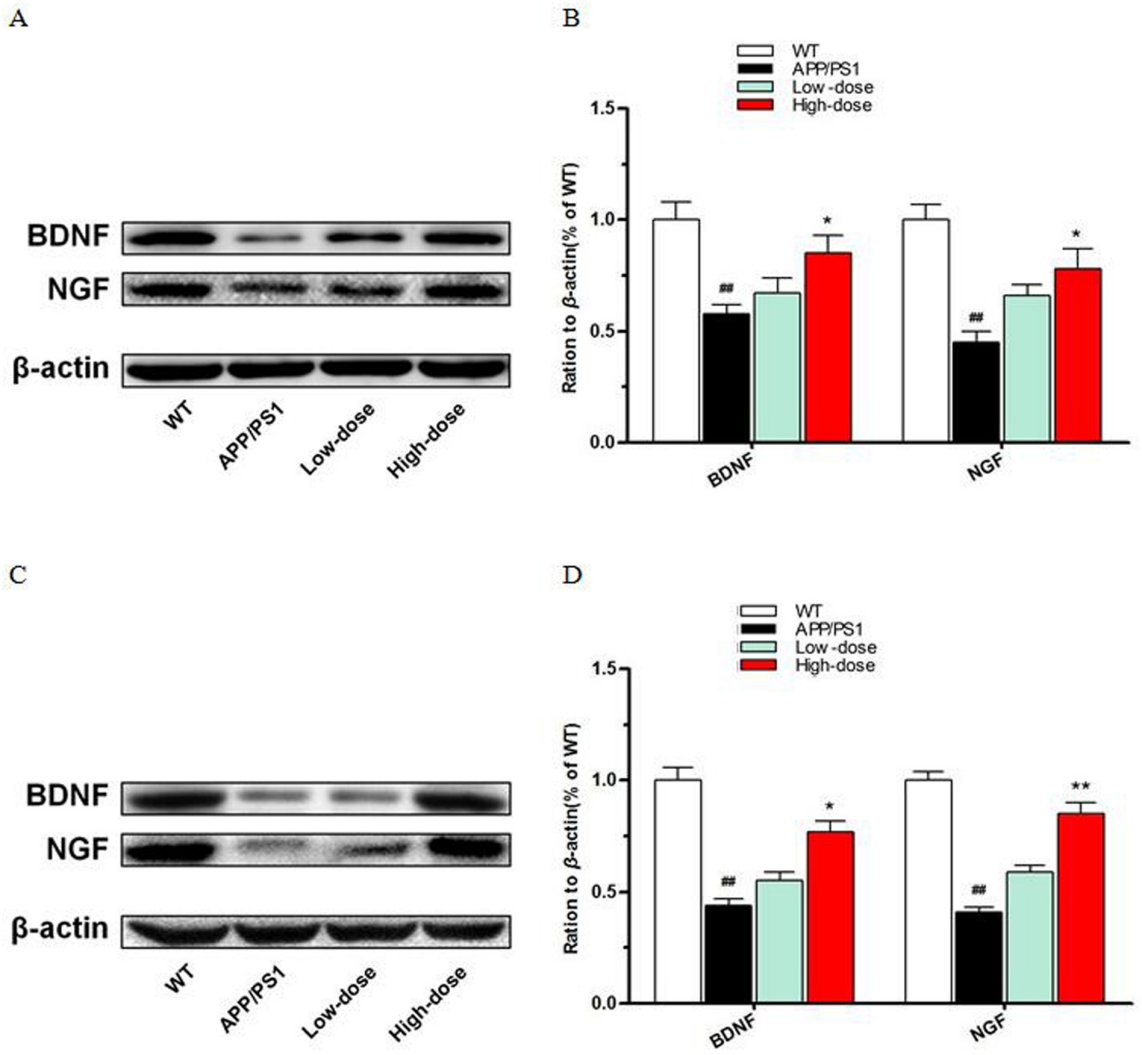
BJJS enhanced the expression level of neurotrophic factors in APP/PS1 mice **(A, B)** The levels of BDNF and NGF in the hippocampus. **(C, D)** The levels of BDNF and NGF in the cortex. Data are shown as the mean ± SEM (n=10 mice per group). ^#^ *P*<0.05 and ^##^ *P*<0.01 versus the wild-type group. ^*^ *P*<0.05 and ^**^ *P*<0.01 versus the APP/PS1 group.

A large amount of research evidence shows that neurotrophic factors are beneficial in reducing the risk of cognitive impairment in neurotrophic systems. What's more, neurotrophic factors could improve synaptic plasticity and promote neuronal survival and function and long-term memory [22]. As shown in Figure 3, we used western blot to detect the expression levels of neurotrophic factors both in the hippocampus and cortex among the 4 experimental groups. Intriguingly, we found that high-dose BJJS significantly increased the expression levels of BDNF (*P*<0.05 vs. APP/PS1) (Figure 3) and NGF (*P*<0.05 vs. APP/PS1) (Figure 3) both in the hippocampus and cortex compared with the APP/PS1 group.

These results suggested that BJJS could enhance the expression of neurotrophic factors in APP/PS1 mice.

### Inhibitory effect of BJJS on neuroinflammation in APP/PS1 mice

**Figure 4:**
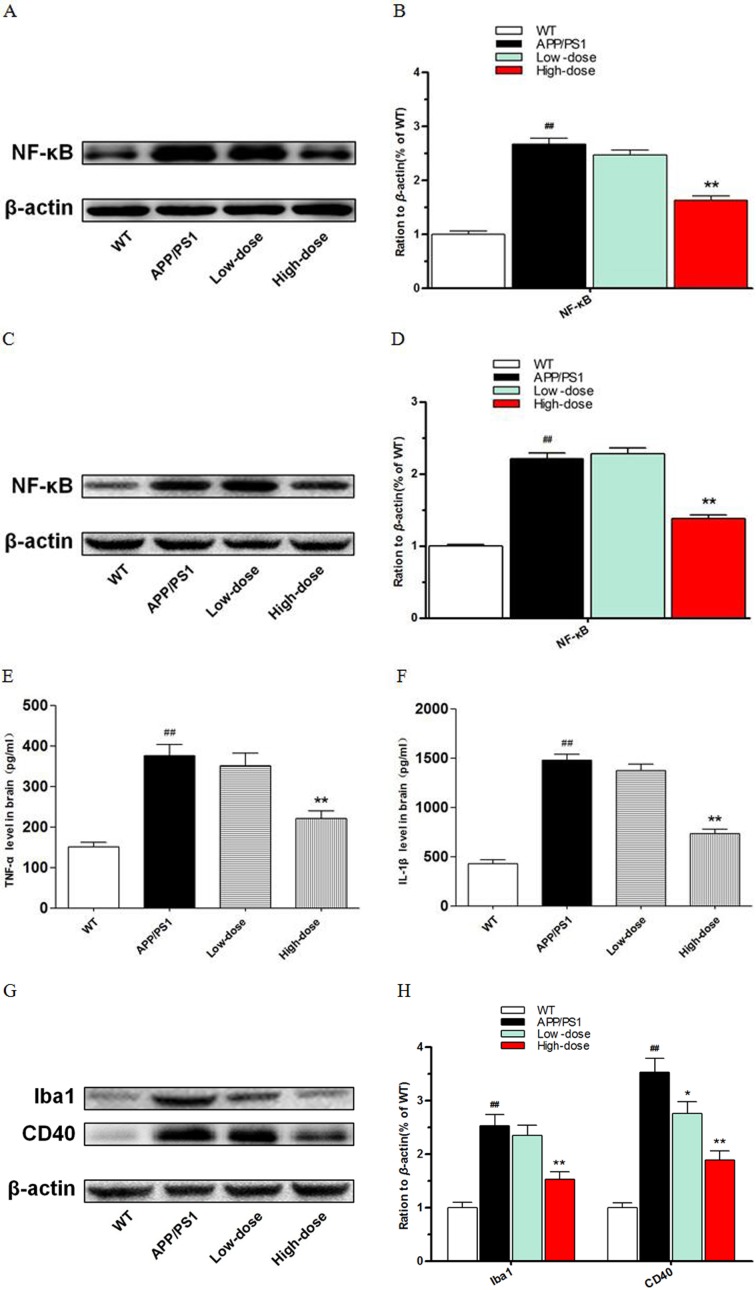
Inhibitory function of BJJS on neuroinflammation in APP/PS1 mice **(A, B)** The levels of nuclear transcription factor-kappa B in the hippocampus. **(C, D)** The levels of nuclear transcription factor-kappa B in the cortex. **(E)** The levels of tumor necrosis factor-α in the cortex. **(F)** The levels of interleukin- β in the cortex. **(G, H)** The levels of microglial markers Iba1 and CD40 in the cortex. Data are shown as the mean ± SEM (n=10 mice per group). ^#^ *P*<0.05 and ^##^ *P*<0.01 versus the wild-type group. ^*^ *P*<0.05 and ^**^ *P*<0.01 versus the APP/PS1 group.

In this study, we found that BJJS had an inhibitory effect on neuroinflammationin APP/PS1 mice. We analyzed the levels of nuclear factor-kappa b (NF-κB), tumor necrosis factor-α (TNF-α), interleukin-1β (IL-1β) and the microglia markers Iba1, CD40 by western blot and ELISA (Figure 4).

NF-κB (*P*<0.01 vs. APP/PS1) (Figure 4A, 4B, 4C, 4D) was found to be reduced both in the hippocampus and cortex of the high-dose BJJS group. TNF-α (*P*<0.01 vs. APP/PS1) (Figure 4E, 4F) and IL-1β (*P*<0.01 vs. APP/PS1) (Figure 4E, 4F) also both remarkably decreased in the cortex of the high-dose BJJS group. What's more, the microglia markers Iba1 and CD40 both significantly reduced in the cortex of the high-dose BJJS group (*P*<0.01 vs. APP/PS1) (Figure 4G, 4H).

### BJJS elevates synapse structure relevant protein in APP/PS1 mice

**Figure 5:**
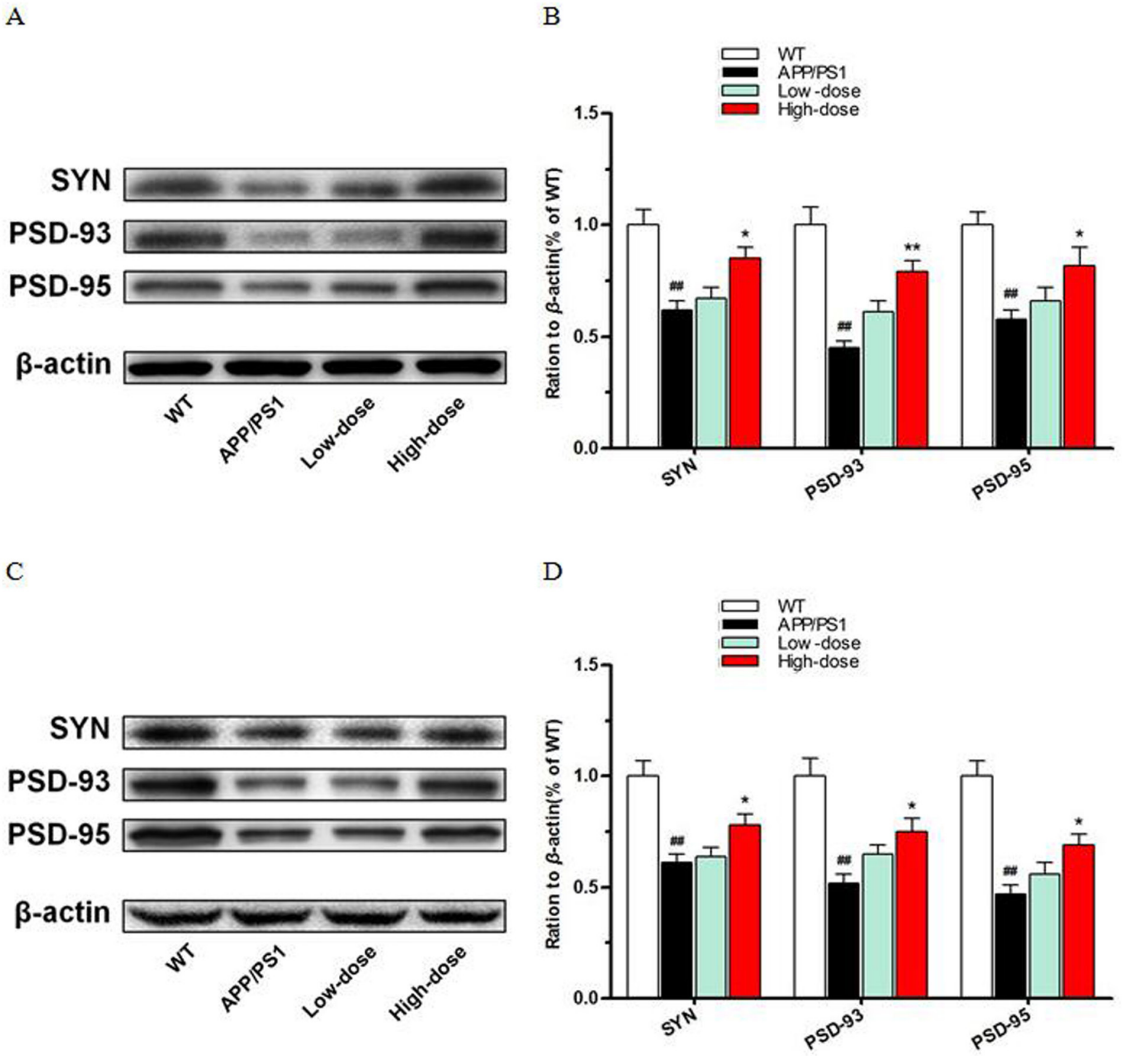
BJJS elevated synapse structure relevant proteins in APP/PS1 mice **(A, B)** The levels of proteins associated with synapse structure stability in the hippocampus. **(C, D)** The levels of proteins associated with synapse structure stability in the cortex. Data are shown as the mean ± SEM (n=10 mice per group). ^#^ *P*<0.05 and ^##^ *P*<0.01 versus the wild-type group. ^*^ *P*<0.05 and ^**^ *P*<0.01 versus the APP/PS1 group.

To evaluate the effect of BJJS on the synaptic structure stability in APP/PS1 mice, we analyzed the protein levels of synaptophysin (SYN), post synaptic density protein-93 (PSD-93), post synaptic density protein-95(PSD-95) by western blot (Figure 5).

As a result, high-dose BJJS increased the levels of SYN (*P*<0.05 vs. APP/PS1) (Figure 5), PSD-93 (*P*<0.01 vs. APP/PS1) (Figure 5) and PSD-95 (*P*<0.05 vs. APP/PS1) (Figure 5) both in the hippocampus and cortex compared with the APP/PS1 group.

As we known, SYN, PSD-93, and PSD-95 play important role in synaptic structure stability. These data showed that BJJS might be beneficial to synapse structure stability in APP/PS1 mice.

## DISCUSSION

In the present research, we demonstrated that BJJS treatment improved the learning and memory abilities and attenuated the progress of AD-associated neuropathological markers in APP/PS1 double transgenic mice. Specific neuroprotective functions of BJJS treatment were observed. Here we summarized the effects of BJJS on APP/PS1 mice: (1) improve the learning and memory abilities, (2) decrease the amount of Aβ oligomers, (3) reduce the number of senile plaques, (4) elevate the levels of neurotrophic factors, (5) inhibit neuroinflammation, (6) is benefit to synaptic structure stability. These results indicated that BJJS may play an important role in the therapeutic of AD. We believe that BJJS attenuated amyloid-β generation and deposition is the basis for improving the learning and memory abilities in APP/PS1 mice and has a close relationship to the reduction of neruroinflammatory factor and the increase of synaptic structure relevant proteins. In addition, BJJS might also play a role in inhibiting neuroinflammation and improving the synaptic structure through regulating neurotrophic factors.

Memory abilities loss, cognitive impairment, and personality changes are the main characteristics of AD in clinical research [23, 24]. At present, APP/PS1 double transgenic mice are one of the most internationally recognized AD animal models [25]. APP/PS1 mice begin to experience spatial learning and memory abilities impairment at the age of 7 months [26, 65]. Here we showed that BJJS treatment alleviated cognitive impairment in APP/PS1 mice via evaluating the learning and memory abilities by the Morris water maze and novel object recognition tests. Our results provide evidence that BJJS relieved the cognitive impairment in APP/PS1 mice.

According to the amyloid-β cascade hypothesis, the accumulation and deposition of amyloid-β are considered to play a key role in the pathogenesis of AD, which is the dominant theory of AD in the past few decades [27, 28]. Under normal physiological conditions, amyloid precursor protein (APP), which secreted by the brain neuronal cells, was cleaved by α-secretase, β-secretase, and γ-secretase. When APP was cleaved by α-secretase, the product sAPPα has a protective effect on neuronal cells. But when APP was cleaved by β-secretase and γ-secretase, the product Aβ1-42 has a strong neurotoxicity. Aβ1-42 is also the main component of the senile plaque. A large number of researches reported that NEP and IDE play critical roles during the pathological process of AD. They can not only degrade extracellular amyloid-β oligomer and destroy the functional domains of amyloid precursor protein, but also degrade and clear amyloid-β in the brain. These functions ameliorate the toxicity of amyloid-β to the CNS [29–33]. APP/PS1 double transgenic mice generate a large number of Aβ and develop a lot of senile plaques both in the hippocampus and cortex during the animal growth period [34]. Our results showed that BJJS treatment decreased the amyloid-β generation and the number of senile plaques in the brain of APP/PS1 mice. Furthermore, we detected a lower level of BACE1, which was one of the amyloid-β generated correlated enzymes, both in the hippocampus and cortex of BJJS-treated group compared with APP/PS1 group. We also found the levels of NEP and IDE, which were amyloid-β degradation correlated proteins, significantly increased both in the hippocampus and cortex of BJJS-treated group compared with the APP/PS1 group. Additionally, the number of senile plaques significantly decreased in the brain of high-dose BJJS group compared with the APP/PS1 group. These results suggest that the level of oligomeric Aβ species was down-regulated by BJJS. Importantly, Aβ oligomers are confirmed to play a key role in synapse toxicity, which is associated with the cognitive impairment [35, 63]. Therefore, BJJS reduced toxic aggregation of Aβ may be the basis for mitigating the cognitive impairment in APP/PS1 mice. Altogether, these data suggest that BJJS treatment can ameliorate pathologic processes of AD induced by amyloid-β.

BDNF is a neurotrophic factor protein synthesized in the brain. It is widely distributed in the central nervous system. During the development of the central nervous system, BDNF acts as a neurotrophic factor for neuronal survival and differentiation, and also plays an important role in neuronal growth and development. What's more, BDNF could prevent neurons from injury and death, improve the pathological state of neurons, and promote the regeneration of injured neurons and other biological effects. It also plays a critical role in mature central, survival of peripheral nervous system neurons and keeping normal Physiological functions [36]. Recently researches have shown that the expression level of BDNF was correlated with the seriousness of the cognitive impairment, and these results suggest that the reduction of BDNF may play an early cofactor in memory abilities loss and cognitive impairment [37]. NGF is also a neurotrophic factor protein that can be found in many animals’ brains, which regulate the growth and development of peripheral and central neurons and maintain neuronal survival. It plays an important role in the central and peripheral neurons development, differentiation, growth, regeneration and functional characteristics of the expression. Recently study shows that NGF could enhance the sprouting of cholinergic neuronal survival and synapse structure stability [38, 39]. Early studies have reported that spatial learning and memory impairment could be reversed by NGF, which means that NGF also plays an important role in the learning and memory abilities [40]. These data demonstrated that BJJS treatment could increase the content of neurotrophic factors in the brain. This effect may play an important part in improving learning and memory abilities in APP/PS1 mice.

In recent years, some studies found that BDNF has a modulatory effect on NF-κB [41, 42]. In addition, there are some reports confirmed that BDNF and NGF have an inhibition effect on neuroinflammation [43–46]. In this study, we found NF-κB down regulated in high-dose BJJS group. And inflammatory factor TNF-αand IL-1βwere also down regulated in high-dose BJJS group.

In the course of many chronic inflammatory diseases, NF-κB was found over activation in the cells, and a large number of NF-κB complexes in the cytoplasm were translocated into the nucleus. This phenomenon induced transcription and expression of the inflammatory mediators and immune-related gene [47]. A large number of amyloid-β protein accumulation will lead to chronic inflammation, damage to neuronal cells. This is an important part of the pathological process in Alzheimer's disease [48]. The clinical study also reported that the levels of TNF-α and IL-1β in blood and cerebrospinal fluid of patients with Alzheimer's disease were significantly higher compared with general population [49, 50]. TNF-α and IL-1β were involved in the immune response and inflammatory response in the body. They can also induce other proinflammatory cytokines and promote the expression of inflammatory-related proteins and play an important role in the amplification of inflammatory cascade [51, 52]. Microglial, act as an immune defense in the central nervous system, are the macrophages of the brain. Microglial cells have the capacity to become activated during various pathological conditions. Due to the shared lineage, many markers are common to both microglia and macrophages, therefore combinations of markers are usually used to identify them. Iba1 is specifically expressed in microglia in the brain and plays an important role in the regulation of the function of microglia [53]. CD40, which is a member of the TNFR family, a costimulatory molecule important for activation of B cells, macrophages, and dendritic cells, is an important molecule that involved in microglial cell activation in brain [54]. In this study, the microglial markers Iba1 and CD40 were significantly reduced in high-dose BJJS group. These results demonstrated that BJJS had an inhibitory function on neuroinflammation in APP/PS1 mice.

A recent study reported that disorders in synapse structure stability appeared earlier to the initial decline of learning and memory abilities than the neuronal cells loss [55]. Synapse loss in hippocampal regions was considered significantly correlated with the severity of cognitive symptom [56]. Then, we also investigated the effect of BJJS on the expression of synapse correlated protein markers. Synaptophysin, a representative presynaptic membrane protein, is primarily present within vesicles. We could evaluate synapse loss by detecting the expression level of SYN [57, 58]. PSD-93 and PSD-95, which both distributed on postsynaptic membranes, are the major scaffolding proteins in the excitatory post synaptic density and serve as potent regulators of synaptic strength. They could not only directly reflect the synaptic morphological changes, but also indirectly reflect the synaptic biological functions. What's more, previous studies showed that the reduction of the levels of synaptophysin, PSD-93 and PSD-95 occurred before the formation of senile plaques in mice with AD [59]. Our results reveal that the levels of synaptic structure stability correlated proteins significantly increased both in the hippocampus and cortex of BJJS-treated group compared with the APP/PS1 group. These results demonstrated that BJJS might be beneficial to synapse structure stability in APP/PS1 mice.

Taken together, our study explored the benefits of BJJS treatment on APP/PS1 mice during the pathological process of Alzheimer's disease from a macro and micro perspective. The results show that BJJS regulated several neuropathological markers correlated with AD, which demonstrated that there were multiple effects of BJJS on the pathogenesis of AD. We consider that the effect of BJJS on the metabolism of amyloid-β may play a central role for improving the learning and memory abilities in APP/PS1 mice. Moreover, BJJS may also play a role in inhibiting neuroinflammation and improving the synaptic structure by regulating neurotrophic factors. All together, our study demonstrated that BJJS may be an effective therapeutic strategy for preventing the development of AD pathology. What's more, in-depth exploration of the mechanism remains to be further studied.

## MATERIALS AND METHODS

### Subjects

The present experiments involving mice were conducted in accordance with policies and procedures described in the Guidelines for the Care and Use of Laboratory Animals published by the National Research Council and was approved by Guangzhou University of Chinese Medicine Animal Ethics Committee.

Genomic DNA of mice was analyzed by tail biopsy using polymerase chain reaction. All of the APP/PS1 transgenic and wild-type mice in this study were obtained from the Model Animal Research Center of Nanjing University. All mice were used at the age of 7 months, include the wild-type mice. Mice of the male were used, weighing 30-35 g, and housed at 20-25°C with 60 % relative humidity under controlled conditions (12h light/dark cycle). Furthermore, the mice had free access to standard rodent diet and water. Behavioral testing was conducted by researchers who were blind to the mice's groups.

### Drugs and treatment

The BJJS was provided by Chinese medicine college of Guangzhou university of Chinese medicine. BJJS was extracted according to the previous reference [20]. BJJS (purity>98 %). Use purified water to dissolve the drug.

In this study, we used 7 months old male mice. APP/PS1 double transgenic mice were randomly divided into 3 groups, APP/PS1, low-dose of BJJS (20mg/kg) and high-dose of BJJS (80mg/kg). The same age non-transgenic mice were selected into wild-type group. 10 mice in each group. Wild-type and APP/PS1 groups were given purified water, 0.01ml·g^−1^, via intragastric administration, once daily for 4 weeks.

After the MWM and NORT tests, the mice were deeply anesthetized with 1% Pentobarbital Sodium and decapitated. The hippocampus and cortex were rapidly dissected on the ice and tissues were immediately flash-frozen in liquid nitrogen.

### Reagents

Rabbit anti-APP (1:4000; Abcam: ab32136), rabbit anti-PS1 (1:1000; Abcam: ab134195), rabbit anti-NEP (1:1000; Abcam: ab126593), rabbit anti-IDE (1:1000; Abcam: ab133561), mouse anti-BACE1 (1:500; Abcam: ab183612), rabbit anti-BDNF (1:1000; Abcam: ab101747), rabbit anti-NGF (1:1000; Abcam:ab68151), rabbit anti-NF-κB (1:1000; Cell Signaling Technology: #8242), rabbit anti-Iba1 (1:1000; Abcam: ab178847), rabbit anti-CD40 (1:1000; Abcam: ab65853) rabbit anti-Synaptophysin (1:1000; Cell Signaling: #4329), rabbit anti-PSD-93 (1:1000; Cell Signaling: #9445), rabbit anti-PSD-95 (1:1000; Cell Signaling: #2507), and mouse anti-β-actin (1:60000; Sigma:A5441). Enzyme-linked immune sorbent assay (ELISA) kits: Mouse amyloid beta peptide 1-42 (15.6pg/mL /1000pg/mL) Emax immunoassay kit (CUSABIO: Lot: R09019069), Mouse TNF-α (6.25pg/mL /400pg/mL) Emax immunoassay kit (CUSABIO: Lot: Y05014334), Mouse IL-1β (62.5pg/mL /4000pg/mL) Emax immunoassay kit (CUSABIO: Lot: X20019335).

### Morris water maze test

The Morris water maze test was performed from day 29 to day 35 after the start of the BJJS treatment. A pool (diameter: 100 cm; height: 50 cm; depth of water: 30cm) was filled with water (22±1°C). A transparent platform (diameter: 8cm; height: 29 cm) was placed at the center of one quadrant in the pool, and the platform was approximately 1cm beneath the water surface, invisible from the surface of the water. The water in the pool was dyed white. During the experiment, some specific graphics were fixed in some place in the wall of the pool to provide additional cues for the mice to locate the platform. Before starting hidden platform experiment, animals receive adaptive training. Mice were released into the water and trained to find the platform within the 60s. In the case of failure, mice were guided to the platform artificially. After finding the platform, mice were kept on the platform for the 20s, and then moved into cage beside an electric radiator until their fur was dried. Each animal accepted 4 times trials every day during the 5 days positioning navigation test. The mice were released into the pool in each trial according to a certain order. The swimming trajectory of each mouse was recorded by a video camera, and a tracking system (Institute of Material Medical, Chinese Academy of Medical Sciences, China) analyzed each mouse's track. The probe trial was performed 24 hours after hidden platform experiment on day 35. The platform was removed. Mice were placed in the pool at the original point with the head toward the wall of the pool and were allowed to swim for the 60s. The mice swimming trajectories within the 60s were systematically recorded and analyzed [60].

### Novel object recognition test

The Novel object recognition test utilizes animals' innate tendency of exploring novel object, which was carried out as previously described [61]. It was performed from day 36 to 38. The test was conducted in an experimental box that was made of opaque plastic (40cm×40cm×45cm). The animals' behavior was recorded by an overhead camera for subsequent analysis. The behavior test is divided into three steps. The testing process consists of adaptation phase, learning phase and testing phase. On the day 36, mice were exposed to an empty experimental box for 5 min to accommodate to the experiment environment. In the learning phase on the day 37, two identical objects were placed on opposite sides of the box. Mice were gently released into the box backing toward the objects for 5min. Before each trial, the box and objects were cleaned with alcohol-based solution (20% w/v) to remove odor cues. Object exploration time was recorded when the mouse touched the object directly with its mouth, forepaws, nose, or vibrissa (within 1-2 cm of the object). In the testing phase on the day 38, one of the objects was replaced with a new one. The preference index was calculated as the time spent on the new object divided by the cumulative time spent on the both two objects.

### Western blot analysis

Tissue was placed in a 1.5 ml centrifuge tube and 10 times the volume of tissue lysate were added, as well as the protease and phosphatase inhibitors followed by homogenization with an ultrasonic shredder. The homogenate was allowed to stand for half an hour at room temperature and then centrifuged at 14000 rpm for 15 min to remove debris and nuclei. The supernatant was boiled for 10 min in a water bath and then put on ice for 5 min; the precipitated proteins were removed by centrifugation 14000 rpm for 5 min. The supernatant was saved for analysis. Use the Bio-Rad DC Protein Assay to determine the protein concentration. Forty micrograms of protein were resolved on 4 to 15% Bio-Rad polyacrylamide gels and then transferred onto polyvinylidenefluoride (PVDF) membranes (Millipore, Germany). The membranes were blocked in Odyssey (LI-COR) blocking buffer for 1.5 hours at room temperature and then incubated overnight in the corresponding primary antibody. After that, the membranes were incubated with corresponding secondary antibodies. Finally, use enhanced chemiluminescent immuno blotting (ECL, Bio-Rad, Japan) to make the membranes visualized. Western Blot quantification was performed using Image lab software.

### Thioflavine-S staining

Thioflavine-S (Th-S), a fluorescent dye, is a common method used to stain senile plaques [62, 63]. The brain tissue of the mice was first fixed with paraformaldehyde, then dehydrated, and embedded in paraffin. The tissues that embedded in paraffin were sectioned with a microtome and fixed on a slide. Th-S staining was carried out in sections as previously described [64–66]. Sections were dehydrating and finally rehydrating in distilled water, and stained with Mayer's hematoxylin for 5 min. After that, Rinse with running water for 1min, immersed in the Th-S solution(1% Ths in distilled water) for 5min. Slices were immersed in 70% alcohol for 5min, and washed with distilled water, 2 times, and finally, glycerol gelatin was used to cover-slipped. All slices were observed blindly by another investigator using an optical microscope(Olympus BX 41 microscope, 40×magnification). Use Image-Pro Plus software to calculate the numbers of senile plaques.

### Enzyme-linked immune sorbent assay

Mouse amyloid beta peptide (Aβ)1-42 in hippocampus and cortex homogenates were quantified using Enzyme-linked immune sorbent assay kits following the manufacturer's instructions as described [67]. The brain tissues were dissociated with 2% sodium dodecyl sulfate. Homogenates suspended were centrifuged at 14000 rpm for 30 min at 4°C. Collect the supernatant fraction for detecting the soluble Aβ1-42. Compare samples with Aβ1-42 standard curves to calculate the level of Aβ 1-42 in extracts.

### Statistical analysis

Statistical analysis software SPSS19.0 was used for data analysis. Data were expressed as Mean±SEM. The escape latency of water maze localization navigation test was analyzed by repeat measurement of variance analysis. Use one-way analysis of variance (ANOVA) for multiple comparisons follow with the Dunnett's post hoc test. S-N-K test was used when homogeneity of variance, and the rank sum test was used when the variance was not uniform. *P*<0.05 was statistically significant.
