# Correction: Neuroprotective effects of bajijiasu against cognitive impairment induced by amyloid-β in APP/PS1 mice

**DOI:** 10.18632/oncotarget.22813

**Published:** 2018-05-22

**Authors:** Haobin Cai, Yijie Wang, Jiayang He, Tiantian Cai, Jun Wu, Jiansong Fang, Rong Zhang, Zhouke Guo, Li Guan, Qinkai Zhan, Li Lin, Yao Xiao, Huafeng Pan, Qi Wang

**Affiliations:** ^1^ Institute of Clinical Pharmacology, Guangzhou University of Chinese Medicine, Guangzhou 510405, China; ^2^ Department of Neurology & Psychology, Shenzhen Traditional Chinese Medicine Hospital, Guangzhou University of Chinese Medicine, Shenzhen 518033, China; ^3^ Guangzhou University of Chinese Medicine, Guangzhou 510405, China; ^4^ Guangzhou Medical University, Guangzhou 510182, China

**This article has been corrected:** The correct 2nd affiliation and author name is given below:

**Haobin Cai**^**2,1**^

^**2**^ Department of Neurology & Psychology, Shenzhen Traditional Chinese Medicine Hospital, Guangzhou University of Chinese Medicine, Shenzhen 518033, China

Original article: Oncotarget. 2017; 8:92621-92634. https://doi.org/10.18632/oncotarget.21515

